# A devolved model for public involvement in the field of mental health research: case study learning

**DOI:** 10.1111/hex.12426

**Published:** 2015-11-16

**Authors:** Pam Moule, Rosie Davies

**Affiliations:** ^1^ Faculty of Health and Applied SciencesUniversity of the West of EnglandStapletonBristolUK; ^2^ National Institute for Health Research Collaboration for Leadership in Applied Health Research and Care West (NIHR CLAHRC West)Faculty of Health and Applied Sciences, People in Health West of EnglandUniversity of the West of EnglandStapletonBristolUK

**Keywords:** case study, community‐based model, mental health, public involvement in research

## Abstract

**Background:**

Patient and public involvement in all aspects of research is espoused and there is a continued interest in understanding its wider impact. Existing investigations have identified both beneficial outcomes and remaining issues. This paper presents the impact of public involvement in one case study led by a mental health charity conducted as part of a larger research project. The case study used a devolved model of working, contracting with service user‐led organizations to maximize the benefits of local knowledge on the implementation of personalized budgets, support recruitment and local user‐led organizations.

**Objective:**

To understand the processes and impact of public involvement in a devolved model of working with user‐led organizations.

**Design:**

Multiple data collection methods were employed throughout 2012. These included interviews with the researchers (*n* = 10) and research partners (*n* = 5), observation of two case study meetings and the review of key case study documentation. Analysis was conducted in NVivo10 using a coding framework developed following a literature review.

**Findings:**

Five key themes emerged from the data; Devolved model, Nature of involvement, Enabling factors, Implementation challenges and Impact. While there were some challenges of implementing the devolved model it is clear that our findings add to the growing understanding of the positive benefits research partners can bring to complex research.

**Conclusions:**

A devolved model can support the involvement of user‐led organizations in research if there is a clear understanding of the underpinning philosophy and support mechanisms are in place.

## Background

Public involvement in the delivery and evaluation of mental health services emerged in the policy landscape in the United Kingdom (UK) in 1990^1^ and continues to be emphasized in 2014^2^ as part of a wider drive to engage service users in shaping service provision.^3^, ^4^, ^5^, ^6^ Furthermore, user involvement in health and social care research in the UK has long been encouraged,^7^, ^8^, ^9^ with provision in place from funding bodies such as the National Institute for Health Research (NIHR), which supports the work of the INVOLVE advisory group.^10^ Inclusion of ‘lived experience’ is argued to improve the quality, relevance, acceptability and ethical status of research^11^, ^12^ but a number of barriers to involvement have also been identified.^13^, ^14^

Interest in how the public are being involved has been emphasized and criteria for the evaluation of involvement in research have been developed,^15^, ^16^ however, the need to measure outcomes of involvement as well as focus on process has been identified.^17^ Initial studies reviewing the involvement of mental health service users in the delivery and evaluation of services reported positive outcomes for both users and researchers,^18^, ^19^ while acknowledging limited engagement and impact on service change at that time. Subsequent research by Perkins and Goddard^20^ explored the impact of a locality‐focused approach to engage local communities in the planning of mental health services in the NHS. This was facilitated in two ways; firstly, through employing a service user as a consultant and link with local independent user groups and other communities and secondly, by funding local community groups to enable their input to the planning of buildings and the care environment. The community groups were able to influence infrastructure development and ultimately impact on the service delivery.

Investigations into the impact of public involvement in research have identified a range of positive outcomes but evidence is often poorly reported^21^ and weak.^22^ Systematic reviews of involvement suggest it has led to benefits for the research design, participants, researchers and wider community organizations.^11^, ^23^ The involvement of community‐based organizations has often been facilitated through staff or members of the organization being involved as representatives of the local community. The reported benefits for community organizations include the ability to develop new knowledge and understanding, an opportunity to raise their local profile to a national level and the potential to form new links with long‐term benefits.^11^ It is also suggested that engagement in research has led to incurred costs and an inability to meet researcher and public expectations.^11^ Both reviews^11^, ^23^ recommend that future studies explore the impact of public involvement in research to develop further understanding of its importance to health‐care research.

This paper presents the results from one case study conducted in the mental health field, as part of a wider research project that evaluated the impact of public involvement in relation to involvement processes across a range of funded research projects.^24^ The objective of this paper is to demonstrate understanding of the processes and impact of public involvement in a devolved model of working with user‐led organizations.

It was one of eight case studies, which included a range of health topics and research projects mainly based in higher education institutions and health‐care providers. This case study was led by a national charity, which used a devolved model of public involvement, where four service user‐led organizations contributed service user perspectives to a multi‐site study. Our research team included one academic and two mental health service user researchers.

## Case study context

The mental health charity had a long history of commitment to public involvement across all of its activities including board level representation of users and carers. It was the only case study in our wider research project^24^ situated in a third sector organization; and was selected as there was evidence of on‐going and active public involvement in the research studies they conducted. Four researchers, including the principal investigator, and three mental health service users from one user‐led organization took part. Our data collection started 1 year into the research case study. Data were collected related to the involvement of one service user‐led organization located in one of the four of the case study sites. The user‐led organization was providing services and support to mental health service users in their local community.

The case study was evaluating the experience and impact of personalization and specifically the use of personal budgets for people with a severe mental illness in four local authorities in England. Personalization necessitates a different way of delivering social care that enables people to be more active in identifying their own support needs and creating tailored packages of support to meet them. It is associated with the provision of direct payments and personal budgets, through which service users are allocated an amount of money to spend on meeting their support needs, according to an agreed plan.^25^ The case study was in its second phase when our data were collected. This second phase consisted of up to three repeat in‐depth interviews with over 50 people receiving social care support through a personal budget, as well as interviews with family members and mental health professionals. The 3‐year case study ran from 2010 until 2013, during which time the research team had changed and the process of working with local user‐led organizations had evolved.

The charity developed a devolved model of working with service user‐led organizations and mental health service users. A key factor in developing this model was the potential to draw on local knowledge of personalization, and build capacity in user‐led organizations. A formal contract was in place between the host charity and four user‐led organizations, which specified their involvement role and financial arrangements. The local organizations had a specific role in providing local intelligence on the implementation of personalized budgets, the recruitment of service user participants for interview and they provided regular individual site reports for the charity. Arrangements were made between the charity and the four organizations and delivered flexibly by individuals within the user‐led organizations.

## Research design and methods

Our wider research project used a case study methodology^26^ which supports the in‐depth exploration of the ‘real‐life’ context. We collected multiple data from the mental health charity led case study between January and December 2012. This included interviews with the researchers and research partners, in addition to accessing key case study documents, such as the protocol and contract. Observational data were collected at two case study team meetings which included participants from all four sites. These meetings concentrated on specific involvement issues and reviewed the site reports. Interviews with researchers were scheduled on three occasions; at the start, the middle and end of the data collection period. Research partner interviews were focussed in one of the four user‐led organizations, selected as this was an active site located in the south‐west, and occurred at the beginning and end of the case study period (see Table 1). These supported access to in‐depth qualitative understanding. In total, 10 interviews were conducted with staff at the charity and five with research partners at the user‐led organization, including all those involved at the selected site. Interviews were guided by a series of questions generated across the wider research project which were adapted to be site specific. Different, but related interview protocols were developed for researchers and research partners at each stage. Later stages included questions related to emerging themes. Initial questions included prior history of involvement; involvement roles, structures and plans; leadership; training and support; and payment. Follow‐up questions addressed involvement activities conducted such as ‘what had gone well?’ and ‘what was more challenging?’; impact of involvement; perceptions of appreciation and value; feedback to research partners; and adequacy of information provision.

**Table 1:** Completed interviews

	1st stage	2nd stage	3rd stage	Total
Researchers	4	4	2	10
Research partners	3	0	2	5

The details of our project (Participant Information Sheet and Consent form) were provided to all participants. Written consent was secured prior to recording observations of the case study team meetings and individual interviews. Interview participants were asked a series of semi‐structured questions informed by the existing literature reviews.^11^, ^23^

All the interviews were recorded, anonymized, transcribed verbatim and analysed within the NVivo10 database. An initial coding framework was developed across the wider research project from the literature review and it was refined at each subsequent stage. Comparison across the transcripts facilitated the identification of overarching themes. The transcripts were coded by PM, who validated the process by cross‐referencing the results of an independent analysis of one script with RD and a second research partner. The two national case study meetings were recorded and reflective notes were made and coded in relation to the identified research themes emerging from the interviews.

All participants were provided with a copy of the report in draft and were invited to comment on the reported data and to highlight any inaccuracies.

Ethical approval was obtained from the National Research Ethics Service, County Durham and Tees Valley Research Ethics Committee, REF No. 11/NE/0251, on the 19 August 2011. An external steering group which included public representation monitored the ethical conduct of the study.

## Findings

Five key themes emerged from the interview data (Devolved model, Nature of involvement; Enabling factors, Implementation challenges, Impact). They are presented and supported by verbatim quotes, which are attributed to either Researcher (R 1–5) or Research Partner (RP 1–3). The stage of interview data collection is noted as S1–3. Observational notes are referenced in support.

## Devolved model

The case study was underpinned by a philosophical position that sought to support local users and user‐led organizations to make a difference locally.

It's partly about empowering people and developing local resource so by doing that of course then those people can learn lots of skills and they can influence their environment…they can become advocates for issues around mental health locally (R3, S1)

Devolving to user‐led organizations was seen as important for gaining local intelligence of personalization, wide local involvement and awareness of changing practices;

They are very effective at providing an on the ground insight…they are involved in local organisational practices and so they keep their ear to the ground in terms of what is going on (R2, S1)

It's about making sure the people have the relevant experience that can influence the study (R3, S1)

The model evolved throughout the case study and became more formalized, to address some of the issues that had emerged, such as a lack of clarity around the role of the user organizations. Contracts were negotiated with user organizations. These were viewed as a ‘live document’ that could be re‐negotiated and amended as the case study progressed. While formal contracts were negotiated between the two organizations, contact was primarily between the research team and the research partners selected as representatives by the user‐led organizations.

We have an agreement with the user organisations to deliver on what's agreed… but the work itself is carried out by those individuals who we directly work with (R1, S1)

We are not employing them [research partners] we are paying the organisations (R1, S1)

## Nature of involvement

The researchers had specific expectations of the role research partners would take in the case study, which had not always been understood in the local organizations. The team felt they could not necessarily expect to recruit experienced research partners in the local sites and had designed the research with this in mind.

…from the very beginning of the design it was never intended that they would be, be interviewing (R3, S1)

…we opted for a model which was more like a research assistant role…helping us in terms of thinking what issues are happening locally, how we can construct interview guides…understand data…help disseminating information that would help with recruitment (R3, S1)

While one research partner had understood this, others had been less clear and wanted a more significant role.

The forum was approached by someone working freelance with [charity] who was trying to recruit user‐led organisations to support …not carry out the research itself but support the participants who might sign up to take part (RP1, S1)

We would have liked it [involvement in interviewing and data collection], yes and I think we would have been capable of doing it as well (RP2, S1)

The above quotes are referring to the employment of a freelance person who, at the start of the case study, sought to encourage user‐led organizations to submit tender proposals to take part. While the user‐led organizations were not collecting data, one of their main roles was to promote the research and secure the recruitment of local service‐users with experience of personalized budgets. The following quotes demonstrate both the researcher and research partner understanding of this;

…key roles are holding an event which is largely about recruitment but also around just promoting the study locally (R1, S1)

Provide support at the recruitment events to explain about the study, try and allay their fears about being involved in research (RP2, S1)

National meetings provided an opportunity for research partner engagement, where two to three representatives for each study site met to present reports, update on activity and any changes in the local landscape relating to personalized budgets. The research partners were expected to represent the user‐led organizations;

We've been asked to give both written reports on a quarterly basis and then to give verbal presentations… of what the latest is in terms of both local work on the [study name] and then any sort of wider developments locally that have got relevance (RP1, S1)

Before they come to the [meeting] they would have a discussion within their local environment… the two people coming can represent not only what those two people think at the time but are actually feeding back these issues (R3, S1)

These meetings also provided a forum to review data collection tools, engage in data analysis and wider case study discussions;

We wanted their advice on the how to word our questions and the flow of the topic guide as well so that's something they helped us with (R4, S1)

They are looking at specific transcripts, looking at two and then meeting and looking at another two, where they are identifying what they think the key things are form those (R1, S3)

## Enabling factors

A strong commitment to public involvement was of key importance to the researchers, evidenced in the ongoing support offered to the research partners. Furthermore, the researchers had provided more targeted assistance, influencing the local authority on one occasion.

Really answering any questions they might have…around recruitment events…day to day things like they would need feedback on specific things that they were preparing…and the financial side of things to do with payments (R4, S1)

We got in touch with the local authority and said just so you know they're [local organisation] struggling to get through to you (R2, S1)

Discussion at the national meeting confirmed research partner views were listened to;

You are positively valuing our lived experience (Observation data)

The research partners also found the national meetings provided opportunities for sharing the difficulties faced by others and welcomed the written agreement that made their role clear.

Supportive to be in that bigger group (RP3, S3)

Not feel so bad about own situation (Observation data)

I think having a written agreement is really good, because it reminds you what their expectations are (RP1, S3)

Financial support was an additional factor that facilitated involvement and was welcomed by the research partners.

We pay them for their travel costs to attend meetings (R5, S2)

You can get your expenses paid for going to the meetings which isn't always the case (RP3, S3)

## Implementation challenges

While the team were intending to support a particular philosophical position through the implementation of the devolved model, it was apparent that tensions emerged challenging the ability of the team to deliver the case study with this conceptual underpinning.

It has gone from something that was very conceptually driven in that there was an idea of the values of it and the you know, what was innovative about it, that drove the original model, and I think that has taken a back seat (R1, S3)

The quote acknowledges the tensions between delivering a user‐led model underpinned by a particular set of values, with the practicalities of completing complex research. Delivery of the case study became the focus and ambitions for local impact became less prominent.

During the course of the case study the team members changed. This affected continuity and resulted in a lack of clarity around case study roles and expectations. For example, members of the team had entered agreements with people at the user‐led organizations which had not been formerly recorded and handed on to their successors.

We had people who had been promised something and then it wasn't necessarily communicated to the rest of the team (R4, S1)

They had some changes of personnel…it did take me a while to get clear what exactly they wanted us to do (RP1, S1)

Maintaining relationships within a devolved model also brought issues. The research partners commented that they would have preferred being more involved in the case study and having more background information. One suggested that they only had access to the documentation written by the user‐led organization such as the local tender document, but had not seen the overall case study plan;

I would like to see something more…that would have been useful (RP2, S1)

The user‐led organizations found it challenging to deliver to some of the contract requirements of the case study. The research partners were unable to implement their planned approach to recruitment. The local authority was unable to offer their support until all necessary documentation from the charity was in place, which obstructed their plans.

…having the local groups was really a way of ensuring we had a different perspective (from that of the local authority) …that hasn't really worked, they have not been in a position to really facilitate recruitment to any extent (R2, S2)

[name] put together a potential framework for recruitment of candidates. He suggested some ideas of things that we could do, advertising in local papers…that was thrown out by [manager name] (RP2, S1)

What was most frustrating was the that everything was taken out of my hands (RP3, S1)

Recruitment was also affected by a reduction in the number of personal budgets being provided locally. Financial constraints were perceived to have impacted on the volume of personal budgets available, with criteria for eligibility being perceived as hard to achieve.

Pretty unsuccessful in terms of directing people to the [study name]… we held two recruitment events but we didn't get any people to sign up through those events (RP1, S1)

They just aren't doing as much of the personalisation in the way that we're interested in researching at the moment (R5, S2)

The existence of a contract, which made clear the expectations of the local organizations, was reported as an enabling factor. However, clarifying these expectations could in some cases lead to research partners feeling under pressure and held to account.

Although having it clearer is very, very useful…I think that once it is in writing there is a danger that groups feel ‘if I don't deliver this I have failed’ (R1, S2)

I did go through a very anxious stage of feeling sort of personally responsible for our failure to recruit people (RP1, S3)

## Impact

It was clear that the research partners had an impact on different aspects of the case study.

The interview guides specifically were changed based on what people said (R3, S1)

Quarterly reports… I think have been useful and they have provided us with a general overview (R2, S2)

The service users were also able to influence data collection and plans for dissemination in the national meetings, with the suggestion below being adopted;

We need examples of good practice to help service users know where to start (Observation data)

The research partners also felt they benefitted personally from their involvement and that there were advantages for the user‐led organizations.

Good to build the relationship with the research team especially for [name] and [name] who are really interested in research (RP1, S1)

It's good for us at a strategic level that we are working on a national project (RP1, S1)

This said, the researchers reported that the research partners were not always informed of the impact they were having in the case study and research partners' comments concurred with this;

I don't know that we do that [provide feedback on impact] very much (R2, S2)

I don't think we have any formalised methods for feeding back (R4, S2)

Nobody has actually said ‘this is what impact you have had’ (RP3, S3)

Research partners were involved in the early analysis of interim findings during the period of data collection, thought this was perceived as limited at this stage with one researcher at the national meeting reporting;

It's just not feasible [to do more] (Observation data)

## Discussion

The study employed an innovative involvement mechanism to benefit both the user‐led organization involved and the individual research partners. This devolved model built capacity and skills within the user‐led organizations. Through this mechanism user‐organizations gained benefit from financial payment and an opportunity to engage in a key national study and work with a large mental health charity. This model of involvement has potential to support increased representation through links to service user led organizations rather than involvement of individuals. Representation is an issue of concern,^27^, ^28^ but there is no agreement about the meaning of this term for involvement. However, in the case study there was no evidence that additional service users had contributed in the site where we collected data.

Researchers were explicit about their underpinning values which is unusual in involvement in research.^29^ Values included both normative values focused on ethics and empowerment, and substantive values^29^ associated with benefits to the research. As well as building capacity the model was intended to support access to local intelligence and experience within study site, help recruitment and provide any support needed for participants to benefit the case study. While this was achieved to some extent, there were issues with local engagement and recruitment, discussed later.

The substantive tasks addressed through involvement in the case study were similar to those identified in reviews of public involvement in research.^11^, ^23^, ^30^ Involvement of the user‐led organizations in the study was organized in a number of ways. Each organization sent two representatives to national meetings, was responsible for submitting local reports on a regular basis and was required to support local recruitment to the case study through organized events and provide support to study participants. While some research partners would have welcomed a greater role in data collection, the research team were clear that this was not an expectation. Attendance at the national meetings facilitated research partner input to developing data collection approaches, tools, analysis of interim findings and informing dissemination plans. However, it was acknowledged that input to the analysis of interim data was minimal for some.

It is recognized that good relationships and positive researcher attitudes within studies are likely to be associated with successful involvement.^15^, ^23^, ^30^, ^31^, ^32^, ^33^ This was evidenced in the case study with all researchers valuing research partner input and being keen to avoid tokenism. Research partners interviewed reported that the meetings were supportive and they felt valued.

Others thinking of using the devolved model would need to take cognisance of the challenges reported here. As with all involvement there were costs as well as benefits.^33^ In taking a devolved approach to involvement the role will be shaped by the local organization, as seen in the case study, as well as being driven by the study requirements. Local variations may be compounded if research staff changes decrease organisational continuity. Additionally, it has been recognized^29^ that tensions can emerge between supporting a user‐led model underpinned by concepts of empowerment and capacity building, and delivering the involvement needed for the study. In this study these tensions were recognized by the researchers.^29^

Contextual factors are increasingly identified as important for involvement,^23^ and Staley^34^ suggests methods to address such issues. The interview data suggested that being embedded in the local context with strong local links had positively impacted the ability of the user‐organization to support involvement in the study. The research partner interviews outlined difficulties in taking their approach to recruitment forward without local authority support and the frustration this had caused. Recruitment was also affected by a reduction in the number of personal budgets being made available locally as a consequence of local financial constraints.

The involvement literature endorses the need for the involvement role to be clarified.^11^, ^16^, ^30^ The development of negotiated contracts between the charity and the local organizations seemed to clarify expectations and roles. However, expectations about recruitment also resulted in one research partner feeling guilty and responsible when recruitment targets were not reached.

Data in the case study corroborates and adds to the evidence base for the impact of involvement supporting the positive influence research partners can have on research.^11^, ^23^, ^33^ The interviews and observation data identified positive impacts in a number of areas, such as the development of research materials and the development of dissemination plans. Research partners commented they had gained personal benefits from supported involvement. Such impacts have also been identified in other studies.^11^, ^23^ It was felt that the user‐organizations had much to gain from their involvement with a national study and developing a collaborative relationship with a national charity. The impact of these relationships over time was not clear within our data collection period, but would be interesting to follow‐up. The case study emphasized the need for mechanisms to feedback about the outcomes of involvement which have been identified by other authors.^28^, ^35^ Although there was reflection on involvement processes in meetings the research partners remained unsure of their impact on the case study.

A number of limitations should be acknowledged when interpreting the results in this paper. Firstly, our data are from one of eight case studies within a larger study. Secondly, the research partner data presented was collected from one of four sites, although there is evidence from the researcher data and observations that some of the reported experiences had resonance across all four sites. Finally, it should be noted that the charity was committed to service user involvement, the research team had an established culture of involvement, and there was clear passion to support this way of working among all of the research staff, which will be have been reflected in the results.

In conclusion, this paper has presented a case study that used an innovative devolved model of involvement. The model demonstrated potential to support capacity building for involvement in research within user‐led organizations, as well as delivering impacts on the research study. This demonstrates that normative^29^ and substantive values^29^ can be combined despite some tensions for researchers The development of a contract helped to clarify expectations, while also placing more explicit pressure to achieve on research partners. Employment of the devolved model required negotiation with the user‐led organizations working peripherally to the research centre.

Despite some of the challenges in the study there was evidence of research partner impact being achieved. It is important to note, however, that it cannot be assumed that those involved will automatically realize the impact they are having, and better communication processes should be developed to ensure research partners receive such feedback and recognize their worth.

## Sources of funding

This research was part of a wider project supported by the UK National Institute for Health Services Research, under its Health Services Research Programme (Reference Number 10/2001/41). Any views and opinions expressed are those of the authors and do not necessarily reflect those of the NHS, the NIHR, the HSR programme or the Department of Health.

## Conflict of interest

None.
